# Machine Learning Methods for Fluorescence Lifetime Imaging (FLIM) Based Label-Free Detection of Microglia

**DOI:** 10.3389/fnins.2020.00931

**Published:** 2020-09-03

**Authors:** Md Abdul Kader Sagar, Kevin P. Cheng, Jonathan N. Ouellette, Justin C. Williams, Jyoti J. Watters, Kevin W. Eliceiri

**Affiliations:** ^1^Department of Biomedical Engineering, University of Wisconsin-Madison, Madison, WI, United States; ^2^Laboratory for Optical and Computational Instrumentation, University of Wisconsin-Madison, Madison, WI, United States; ^3^Department of Comparative Biosciences, University of Wisconsin-Madison, Madison, WI, United States; ^4^Morgridge Institute for Research, Madison, WI, United States

**Keywords:** FLIM, CNS, machine learning, brain metabolism, neural networks, microglia, NAD(P)H

## Abstract

Automated computational analysis techniques utilizing machine learning have been demonstrated to be able to extract more data from different imaging modalities compared to traditional analysis techniques. One new approach is to use machine learning techniques to existing multiphoton imaging modalities to better interpret intrinsically fluorescent cellular signals to characterize different cell types. Fluorescence Lifetime Imaging Microscopy (FLIM) is a high-resolution quantitative imaging tool that can detect metabolic cellular signatures based on the lifetime variations of intrinsically fluorescent metabolic co-factors such as nicotinamide adenine dinucleotide [NAD(P)H]. NAD(P)H lifetime-based discrimination techniques have previously been used to develop metabolic cell signatures for diverse cell types including immune cells such as macrophages. However, FLIM could be even more effective in characterizing cell types if machine learning was used to classify cells by utilizing FLIM parameters for classification. Here, we demonstrate the potential for FLIM-based, label-free NAD(P)H imaging to distinguish different cell types using Artificial Neural Network (ANN)-based machine learning. For our biological use case, we used the challenge of differentiating microglia from other glia cell types in the brain. Microglia are the resident macrophages of the brain and spinal cord and play a critical role in maintaining the neural environment and responding to injury. Microglia are challenging to identify as most fluorescent labeling approaches cross-react with other immune cell types, are often insensitive to activation state, and require the use of multiple specialized antibody labels. Furthermore, the use of these extrinsic antibody labels prevents application in *in vivo* animal models and possible future clinical adaptations such as neurodegenerative pathologies. With the ANN-based NAD(P)H FLIM analysis approach, we found that microglia in cell culture mixed with other glial cells can be identified with more than 0.9 True Positive Rate (TPR). We also extended our approach to identify microglia in fixed brain tissue with a TPR of 0.79. In both cases the False Discovery Rate was around 30%. This method can be further extended to potentially study and better understand microglia’s role in neurodegenerative disease with improved detection accuracy.

## Introduction

Unlike external fluorescent labeling approaches, label-free microscopic identification methods can provide equally useful information while leaving the cellular microenvironment unperturbed. Identification of unique metabolic fingerprints based on quantitative data obtained from endogenous cellular properties has been recently explored to develop biomarkers of different cell types and/or disease states. These techniques take advantage of different optical imaging modalities and the intrinsic properties revealed by them followed by quantification techniques to identify different biomarkers. Examples include: diffused optical tomography for breast cancer (Flexman et al., 2013), collagen signature (Kirkpatrick et al., 2006), Stimulated Raman Scattering (SRS) based label-free chemical contrast (Freudiger et al., 2008), and fluorescence lifetime based macrophage signature (Szulczewski et al., 2016).

Fluorescence Lifetime Imaging Microscopy (FLIM) is a well-suited modality for identifying candidate biomarkers as it can be used to assess intrinsic cellular metabolism. Fluorescence lifetime depends on physiological parameters such as pH and ion/oxygen concentrations; it is also independent of intensity, concentration, sample absorption, and sample thickness (Berezin and Achilefu, 2010; Suhling et al., 2015). FLIM can monitor metabolism by taking advantage of the intrinsic fluorescence of the ubiquitous metabolic coenzyme NAD(P)H (Nicotinamide Adenine Dinucleotide). NAD(P)H is a key electron donor/acceptor involved in many metabolic processes, especially redox reactions (Lakowicz et al., 1992; Skala et al., 2007; Mongeon et al., 2016). FLIM can quantify the ratio between free and bound NAD(P)H, and calculate the mean fluorescence lifetime based on the relative quantity of free: bound components and the individual component’s lifetime (Lakowicz et al., 1992; Bird et al., 2005; Skala et al., 2005; Provenzano et al., 2008). The mean lifetime, long lifetime component, or free: bound ratios of NAD(P)H are indicative of whether a cell’s metabolism is in a more glycolytic or oxidative state. For example, more free NAD(P)H measured via FLIM can be used to show a shift toward glycolysis in cancer per the Warburg theory. As a result, FLIM is gaining widespread acceptance as a way to probe the cellular microenvironment (Wang et al., 1992; Suhling et al., 2005, 2015; Provenzano et al., 2008; Berezin and Achilefu, 2010). FLIM is also increasingly used to probe brain metabolism and neuronal function *in vivo*. For instance, quantitative FLIM data has been used by researchers to (1) find contrast between glioblastoma and normal brain tissue (Leppert et al., 2006; Sun et al., 2010; Kantelhardt et al., 2016), (2) map alterations in cerebral metabolism based on NAD(P)H binding (Chia et al., 2008; Yaseen et al., 2017), (3) non-invasively, optically image Alzheimer’s Disease (Das et al., 2018), (4) visualize redox activities in the brain (Mongeon et al., 2016), and (5) quantify neuronal dysfunction in neuroinflammation using FLIM instrumentation (Rinnenthal et al., 2013).

One type of cells of growing imaging interest has been those of the central nervous system (CNS) such as microglia. Microglia are a critical cell type in the nervous system whose activities are implicated in virtually all neuropathologies including traumatic injury, neurodegenerative disease, ischemia, and infection (Watters et al., 2005; Garden and Möller, 2006; Tambuyzer et al., 2009; Charles et al., 2011). These CNS tissue-resident macrophages influence brain development, maintain the neural environment, respond to injury and infection, and orchestrate repair processes among other important functions. Their production of neurotoxic inflammatory molecules often exacerbates neuronal damage. Due to this biological significance and the need for improved tools for further characterization, we chose microglia as our biological use case for developing a non-invasive label free imaging workflow that combines fluorescence lifetime imaging with machine learning.

The goal of this study was to develop a FLIM-based fingerprint for microglia, as has been done for macrophages (Alfonso-García et al., 2016) and bacteria (Bhattacharjee et al., 2017). Our lab previously demonstrated that FLIM-based, label-free imaging could be used to distinguish unique glycolytic type FLIM signatures between tumor-associated macrophages and mouse mammary tumors cells (Szulczewski et al., 2016). However, this has not been done in the context of the CNS and microglia. Previous studies have used FLIM to show neural stem cell differentiation (Stringari et al., 2012) or characterize the metabolic response of astrocytes (Stuntz et al., 2017), but not to identify microglia identity. In order to optimally exploit differences in metabolism-induced lifetime changes for microglia identification, a fast, quantitative approach that can identify their unique metabolic signature would be ideal. Accordingly, we have coupled FLIM with a machine learning-based solution to detect microglia based on their FLIM parameters.

One of the most common ways FLIM acquisition is performed is Time Correlated Single Photon Counting (TCSPC) connected to multiphoton laser scanning microscopes. TCSPC offers picosecond level time resolution, which is arranged in histograms based on timing information of the detection photon. In a typical time domain FLIM analysis workflow, the lifetime decay curve is subject to one- or two-component exponential curve fitting to estimate the lifetime parameters such as free: bound NAD(P)H lifetime, the amplitude of their decay curve, and goodness of fit (chi-squared error). If a machine learning (ML) algorithm has raw unfitted decay data, it could be possible to estimate lifetime accurately and subsequently characterize cell types. This approach would be advantageous in two ways: (i) it will reduce time for calculating lifetime from a decay curve (the only remaining bottleneck would then be collection time; the lifetime can be estimated instantly without the time-consuming exponential curve fitting); and (ii) possibly identify an optical/metabolic fingerprint which would otherwise not be apparent with regular analysis. This approach of calculating lifetime directly from the decay curve is relatively new, to our knowledge, there is one previous published report that has estimated lifetimes using artificial neural networks (Wu et al., 2016). Another approach of possible utility would be to use the estimated lifetime parameters calculated using standard curve fitting approaches and train where the label is created by antibody staining. Recently there is work by Smith et al. (2019) have utilized deep neural network with FLIM data to estimate lifetime in fit free fashion. Another group demonstrated machine learning approach using random forest classifier on FLIM data for classification of tumor FLIM image (Unger et al., 2020).

In this study, we have used both approaches to train an Artificial Neural Network (ANN) to identify the location of microglia, i.e., (i) a fitting based method (FBM) where the fitted data are exported from standard curve fitting routines, and (ii) an experimental decay based method (DBM) training with the exponential decay curve consisting of 256 time-bins directly. To our knowledge, this is the first application of a FLIM-based machine learning application for intrinsic fluorescence signature development.

## Materials and Methods

### Animals

All animals were maintained in an AALAC-accredited animal facility with a 12-h light/dark cycle regime and had access to food and water *ad libitum*. All experiments were performed in accordance with the University of Wisconsin-Madison Institutional Animal Care and Use Committee.

For FLIM imaging, 100 μm thick coronal slices were prepared from the fixed brains of young adult C57BL/6J and CX3CR1-GFP mice (Jackson Labs), aged 6–8 weeks. Animals were euthanized by isoflurane overdose and transcardially perfused with ∼30 ml of ice-cold PBS, followed by a second perfusion with an ice-cold solution of 4% PFA in PBS. Brains were then dissected, post-fixed for 24 h in a solution of 4% PFA in PBS, and then moved to HBSS (all performed at 4°C and protected from light).

### Preparation of Primary Neonatal Mixed Glial Cultures

Mixed primary glial cultures were prepared from 3 to 7 day old, CX3CR1-GFP mouse pups as previously described (Crain et al., 2013). Briefly, brains were dissected immediately after decapitation, and the brain stem, olfactory bulbs, meninges, and visible blood vessels were removed. The remaining tissue was finely minced, thoroughly triturated with a serological pipette in 0.25% trypsin-EDTA containing 0.1 mg/ml deoxyribonuclease I, and then incubated at 37°C for 20 min. The reaction was immediately stopped by the addition of an equal volume of heat-inactivated horse serum. The dissociated cells were resuspended in DMEM supplemented with 10% FBS and 100 units/ml penicillin/streptomycin. Brains were processed individually for each pup, and the resulting cell suspension was divided equally and plated in 35 mm dishes (4–6 plates/brain). The plated cells were cultured for 7–14 days in a 37°C incubator supplemented with 5% CO_2_; the culture medium was replaced every 3–4 days.

### Immunohistochemistry

One hundred micrometers thick coronal sections were prepared from the midbrain region of each brain using a Leica Vibratome. Two slices from each animal were used for immunohistochemical staining. Briefly, slices were washed at room temperature with 0.3% TritonX-100 in PBS, before incubating in blocking buffer (1% BSA, 0.3% TritonX-100/PBS) for 2 h at room temperature. Slices were then incubated with anti-Iba1 antibodies (1:1000; Wako Catalog No. 019-19741) in blocking buffer in the dark at 4°C overnight. Slices were washed again at room temperature with 0.3% TritonX-100 in PBS followed by incubation in the dark for 2 h with AlexaFlour594 anti-rabbit IgG antibodies (1:200) in blocking buffer, at room temperature. Slices were washed with 0.3% TritonX-100 in PBS and mounted on 1 mm slides using Cytoseal60 mounting medium. Mounted sections were stored at room temperature, protected from light until they could be imaged.

### Multiphoton Lifetime Imaging

The multiphoton based (Denk et al., 1990) lifetime and intensity imaging was performed on a custom multiphoton laser scanning system built around an inverted Nikon Eclipse TE2000U at the Laboratory for Optical and Computational Instrumentation (Yan et al., 2006). A 20× air immersion objective (Nikon Plan Apo VC, 0.75 NA) (Melville, NY, United States) was used for all imaging. For NAD(P)H imaging, data was collected using an excitation wavelength of 740 nm, and the emission was filtered at 457 ± 50 nm (Semrock, Rochester, NY) for the spectral peak for NAD(P)H/NADPH. For GFP intensity imaging, the excitation was set at 890 nm, and an emission 520 ± 35 filter was used (Semrock, Rochester, NY). For AlexaFluor594 imaging, excitation was set at 810 nm, and a 615/20 (Semrock, Rochester, NY) bandpass emission filter was used for emission. We used time domain FLIM imaging where the FLIM decays curves were built with TCSPC (Time Correlated Single Photon Counting) electronics. FLIM images of 256 × 256 pixels were collected with 120 s collection using SPC-150 Photon Counting Electronics (Becker & Hickl GmbH, Berlin, Germany) and Hamamatsu H7422P-40 GaAsP photomultiplier tube (Hamamatsu Photonics, Bridgewater, NJ). Urea crystals were used to determine the Instrumentation Response Function (IRF) with a 370/10 bandpass emission filter (Semrock, Rochester. NY). For each sample, around 20 neighboring FOVs were randomly selected, and the average value of lifetime and free NAD(P)H ratio was calculated based on masking described in the “Data Analysis” section. The instrument response function of the optical system was calibrated during each imaging session. Autofluorescence intensity and fluorescence lifetime data were analyzed in SPCImage (Becker & Hickl GmbH, Berlin, Germany) where a Levenberg–Marquardt routine for nonlinear fitting was used to fit the fluorescence decay curve collected for each decay after binning. Data were assessed by the minimized chi-square value generated during the fit so that the analyses were unbiased. To eliminate background fluorescence, a threshold for analysis was applied based on photon counts.

### Data Analysis

**FIGURE 1:**
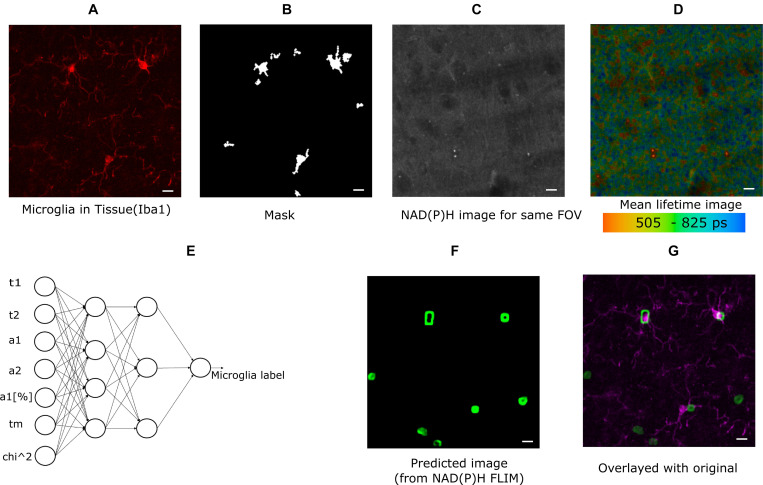
Method demonstrating microglia prediction using ANN. **(A)** Microglia image using Iba1. **(B)** Mask created from Iba1 image **(C)** NAD(P)H intensity image from the same FOV. **(D)** Lifetime image of the same FOV created from SPCImage (Becker and Hickl GmbH, Berlin, Germany) **(E)** ANN showing the inputs used for training instances and output **(F)** predicted microglia image. **(G)** Composite image showing fusion of predicted microglia image and actual microglia image created from Iba1 (scale bar 10 μm, **A–D,E,F**).

The cell cultures used for experiments were CX3CR1-GFP positive, and the GFP intensity image was used to create the mask for identifying microglia. For the brain tissue imaging, anti-Iba1 antibodies visualized with AlexaFluor594 was used to identify microglia (Figure 1A) and the mask is shown in Figure 1B. The lifetime fitted data from SPCImage (Becker & Hickl GmbH, Berlin, Germany) and all the parameters were exported for more custom operations. Figure 1C shows the NAD(P)H intensity image created from the lifetime image. Figure 1D shows the lifetime image (mean lifetime) created by curve fitting. Mean lifetime is one of the parameters exported for ANN training. The exported data were imported in MATLAB (MathWorks Inc., Natick, MA) for calculating the means of custom regions and statistical analyses. The masks were created from intensity images in MATLAB. The time-resolved Becker and Hickl SDT data were read using Bio-Formats (Linkert et al., 2010) MATLAB support package. For generating the threshold of the predicted image, we tuned a optimal threshold that maximizes overlap on the training images. Then the trained network is applied to the test images and the threshold is applied. A cell is considered a positive detection when it overlaps with the cell body or the processes, otherwise it is considered a false positive.

#### ANN Implementation, Fitting-Based Method (FBM)

In this approach, the fitted data for NAD(P)H lifetime was exported in ^∗^.asc format from SPCImage and read in MATLAB for post-processing and training. The exported data were mean free/bound/mean NAD(P)H lifetimes (τ_1_, τ_2,_ τ_m_), amplitude of their decay curve and free bound ratio (a_1,_ a_2,_ a_1_[%]), goodness of fit (chi-squared error, χ2). The neural network for training and testing is applied on the image data after some preprocessing to create smaller block. All exported images were exported from 256 × 256 size lifetime images, and smaller overlapping blocks of 8 × 8 size were taken from each dataset for training instances; the average of each block was calculated for each parameter. The neural network is applied on the each pixel of the new calculated point from the 8 × 8 block. In this way, 8,494,137 labeled training datasets were created for cells, and 5,456,088 training datasets were created for tissue training. Subsequently, the data was used for training using MATLAB’s neural fitting toolbox. Seventy percentage of the dataset was used for training, and Fifteen percentage was set aside for validation and testing. Bayesian regularization (Foresee and Hagan, 1997) backpropagation was used as a network training function, that updates the values according to Levenberg-Marquardt optimization. We used a feed-forward network with one hidden layer with 10 neuron in the hidden layer. As a cost function, we used mean squared error. The performance index is the mean square error, generated by the neural network toolbox comparing the result of test and training dataset (random splitting between training/testing/validation) with a number ranging from 0 to 1, 0 indicating maximum match 1 indicating no match. The performance with 10 hidden neuron and Bayesian Regularization was 0.0202 for mixed glial cell culture and 0.013 for tissue; increasing the number of hidden neurons further did not improve performance. For example, with 20 hidden neuron with cell the performance was 0.020 but with five hidden neuron the performance was 0.024. With additional hidden layers, the performance remained the same. We also compared training performance using Naive Bayes classifier, support vector machine (SVM) and K-nearest neighbor (Knn). We reached performance of 0.92 with Naïve bayes and 0.802 with Knn. With SVM the performance varied from 0.07 to 0.15. Given the performance achieved by ANN, we decided to used it for further classification. The accuracy for microglia detection was determined by first exporting the FLIM parameters from NAD(P)H lifetime from a separate testing dataset. The input data for feeding the ANN (Figure 1E) was created the same way as the training process. Following this step, the label for each block was predicted using the trained model, and the label for that block was created. Repeating this step for all pixels generated a 2D image with microglia detection probabilities. Pixels with lower probability was discarded and smaller detected regions were also discarded. The calibration was done on training images to ensure overlap. Then, these thresholds were used to create a probability image. Figure 1F shows such a mask which has seven microglia in the FOV. The predicted image was compared with antibody-labeled (Figure 1A) images to locate microglia positions. The composite image (Figure 1G) shows the overlap between the predicted microglia region and antibody detected microglia region. We calculated the sensitivity (True Positive Rate), Positive Predictive Rate (PPV), False Negative Rate (FNR), and False Discovery Rate (FDR) (Sammut and Webb, 2017) from the detected microglia after prediction and post-processing. We did not calculate the rest of the parameters of the confusion matrix as we do not have the true negatives with our current approach of imaging. We do not have labeled data for non-microglia cells, as it was out of the scope of this paper.

#### ANN Implementation, Decay-Based Method (DBM)

This approach exports the decay directly, which has 256 times bins in the histogram. The data were directly read from time-resolved SDT file using the Bio-Formats file reader library (Linkert et al., 2010) in MATLAB. Instead of using the fitted parameters as input to the ANN, we used an ANN with 10 hidden neuron and 256 input nodes. The rest of the training and testing was the same as the previous method. The performance with these settings for cells was 0.0227.

## Results

### ANN-FLIM Can Detect Microglia in Mixed Glial Cell Cultures

**FIGURE 2:**
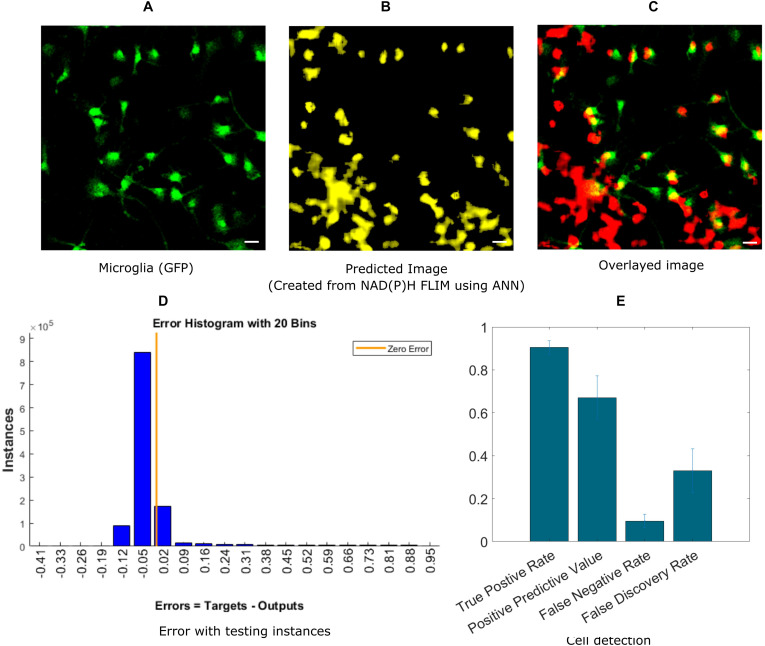
Prediction of microglia from NAD(P)H lifetime data in mixed glial cell cultures where microglia cells are GFP-labeled. In this approach, ANN is applied on exported lifetime parameters of the endogenous fluorophore NAD(P)H. **(A)** Original fluorescence intensity image of microglia created from the GFP channel **(B)** Predicted microglia image from NAD(P)H lifetime data from the same field-of-view. **(C)** Composite image of predicted microglia image and original intensity image. Most of the microglia are accurately predicted as seen from the composite image. But there are a few microglia not detected and some false positive in the lower left corner **(D)** Error rate from testing instances, created from the testing microglia dataset **(E)** TPR, PPV, FNV and FDR from five different dishes (Scale bar 20 μm, **A–C,E**).

In this section, we demonstrate the ability of ANN-based techniques to identify microglia in mixed glial cell cultures. ANN is applied on exported data after curve fitting from SPCImage software. NAD(P)H lifetime fitted data are exported from SPCImage (see section “Materials and Methods”), and exported parameters are used to compute training instances for individual blocks. The training is performed on training sets and tested on a separate testing dataset. Figure 2A shows the GFP intensity image created from GFP positive microglia. The predicted microglia image from a sample field of view is shown in Figure 2B. Figure 2C shows the fused image of the original microglia image and predicted image from the same FOV. It is evident from the fused image that, most of the microglia are properly detected when compared with actual microglia image created from GFP. There are some microglia which are not predicted and some false positive in the lower left corner where microglia are falsely identified. Figure 2D shows the error obtained by prediction of individual instances created from FLIM parameters of the testing dataset. The total number of microglia in all (testing) FOVs were 348 and of them 313 were correctly identified, 35 microglia was missed by the prediction algorithm, but 138 additional microglia was falsely identified. Figure 2E shows the result for microglia detection for five different dishes; we got TPR 0.90 ± 0.03, PPV 0.67 ± 0.10, FNR 0.09 ± 0.03, FDR 0.33 ± 0.10.

### ANN-FLIM Can Detect Microglia in Brain Tissue

**FIGURE 3:**
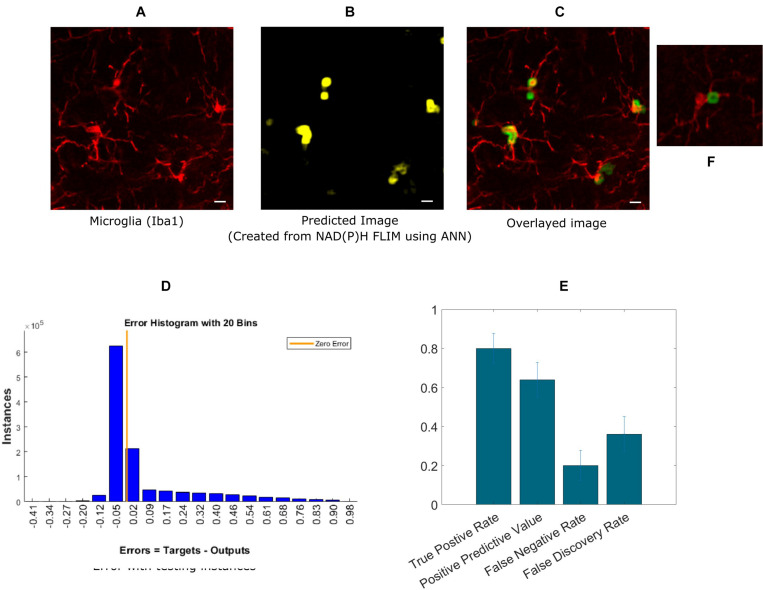
Prediction of microglia from NAD(P)H lifetime data in mouse brain tissue. In this approach, ANN is applied on exported lifetime parameters of the endogenous fluorophore NAD(P)H. **(A)** Original intensity image of microglia location created from the anti-Iba1 antibody AlexaFluor594 channel **(B)** Predicted microglia image from NAD(P)H lifetime data from the same field-of-view. Composite image of predicted microglia image and original intensity image. **(C)** Composite image of predicted microglia image and original intensity image. **(D)** Error rate from training and **(E)** TPR, PPV, FNV, and FDR from five different tissues (Scale bar 10 μm, **A–C,E**). **(F)** Zoomed in image of a microglia where a process is identified as positive.

Next, we extended our approach to identifying microglia in fixed mouse brain tissue slices. Imaging brain tissue is more challenging than imaging cell cultures because of greater heterogeneous spatial structures, and a wider degree of variation in microglia lifetimes. The algorithm implementation was the same as in the *in vitro* cell culture experiments, where ANN was applied to the exported lifetime data from curve fitting software, SPCImage. An anti-Iba1 antibody with an AlexaFluor594 secondary antibody was used to visualize and create a microglial intensity image (Figure 3A). Figure 3B shows the predicted microglia image created from NAD(P)H lifetimes of the same field of view as in Figure 3A, in which the microglia are stained with Iba1. Figure 3C shows the fused image of the original microglia image (from tissue)- and predicted image from the same FOV. It is evident from the fused image that all of the microglia from this FOV is properly detected when compared with the Iba1 intensity positive microglia. Figure 3D shows the error obtained by while predicting using individual instances created from FLIM parameters of the testing dataset. The total number of microglia in the testing FOVs are 170 and 137 were correctly identified, but 76 microglia were falsely identified. Figure 3E shows the result for five different tissues where we got TPR 0.79 ± 0.08, PPV 0.638 ± 0.09, FNR 0.2 ± 0.08, FDR 0.36 ± 0.09. The TPR is reduced for microglia in tissue and FNR is increased as the heterogeneity and complexity of the structure complicates the prediction.

### ANN Directly on Exponential Decay Can Detect Microglia in Mixed Cell Cultures

**FIGURE 4:**
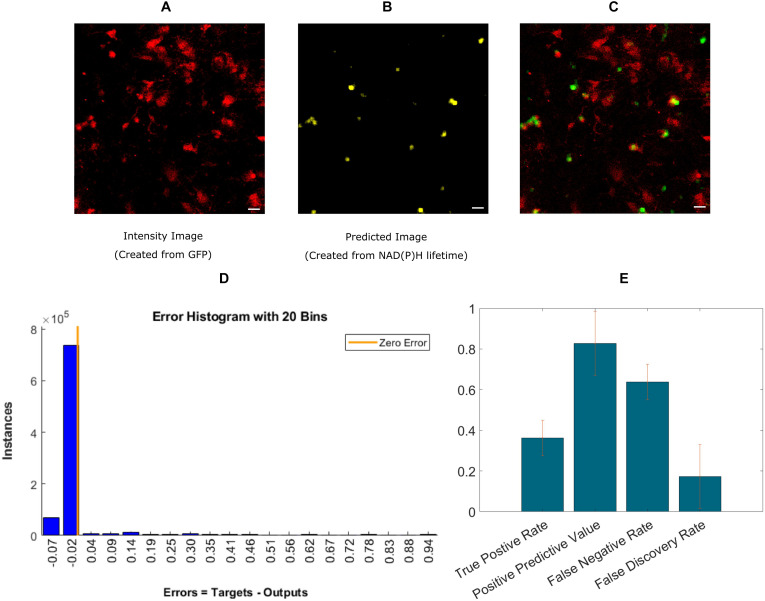
Prediction of microglia from NAD(P)H lifetime data in mixed glial cell cultures where microglia cells are GFP-labeled. In this approach, ANN is applied directly on exponential decay curves of the endogenous fluorophore NAD(P)H. **(A)** Original intensity image of microglia location created from the GFP channel **(B)** Predicted microglia image from NAD(P)H lifetime data only. Both images are from the same field-of-view. **(C)** Composite image of predicted microglia image and original intensity image. **(D)** Error rate from training and **(E)** TPR, PPV, FNV, and FDR from five different dishes (Scale bar 20 μm, **A–C**).

Finally, we implemented an experimental approach, where instead of exporting the lifetime fitted data, we used the decay data having 256-time bins as a training instance. A recent study showed the effectiveness of using ANN to calculate lifetime directly from decay (Wu et al., 2016). Instead of calculating lifetime, we directly used microglia locations as labels. This approach is simpler because it bypasses the steps involving exponential curve fitting routines and exporting the fitted lifetime parameters. Figure 4A shows the intensity image of microglia created from the GFP channel in the mixed glial cultures. Figure 4B shows the recreated image from ANN-predicted microglia from the exponential decay. Figure 4C shows the fused image of the original microglia image (from mixed glial culture)- and predicted image from the same FOV. Figure 4D shows the error obtained by while predicting using individual instances created from FLIM parameters of the testing dataset. The total number of microglia in the testing FOVs are 371 and 136 were correctly identified, but 24 microglia were falsely identified. Figure 4E shows the result for five different dishes where we got TPR 0.36 ± 0.09, PPV 0.82 ± 0.16, FNR 0.63 ± 0.08, FDR 0.17 ± 0.15.

## Discussion

In this paper, we have used ML and fluorescence lifetime to identify microglia in both mixed cell cultures and in brain tissue sections. To our knowledge, this is one of the first studies to apply ML algorithm to FLIM data to identify intrinsic cellular metabolic signatures. Moreover, applying ML methods directly on exponential decay data can be potentially augmented to calculate lifetime without curve fitting to help understand underlying trends in biological samples. The techniques applied in this paper can be extended in various ways in studies related to brain metabolism. Although there have been some FLIM studies to visualize brain metabolism, but ML-FLIM was not used to characterize cell types previously. This technique can be potentially extended to identify other CNS glial cells such as astrocytes, oligodendrocytes, and neurons. For *in vivo* brain studies, a ML-based, label-free technique can be used in the future where FLIM for different spectral channels can be used in conjunction with trained ML networks to identify and study specific cell types. Here, we used the lifetime information generated by exponential curve fitting from each pixel as training instances, and antibody-stained microglia locations are used as positive microglia pixels. The goal was to be able to identify microglia using the NAD(P)H lifetime information alone. We have used two different approaches for training, (1) the calculated lifetime parameters created from curve fitting software SPCImage, FBM, and (2) ML directly on time-resolved data, DBM. The second approach bypasses the time-consuming curve fitting process and reduces the overall number of processing steps. We first implemented our ANN-based approach on microglia in mixed glial cell culture and then extended this to testing tissue samples. In the future, the tissue-based identification can be repurposed for *in vivo* identification of microglia given there was enough training data before detection testing.

Microglia are normally visualized with antibody-based methods, using antibodies, such as anti-TMEM119 (Bennett et al., 2016), anti-Iba1, and the combination of anti-CD11b and anti-CD45, among others. These standard labeling techniques have several limitations, including the extended sample preparation time and associated complexity, inefficient antibody penetration, and the potential for non-specific antibody binding. A FLIM based, label-free imaging technique is advantageous because it is simple, free from exogenous labeling, provides the ability to directly measure intrinsic cellular properties, and its potential for extending its use to observe cell activity *in vivo*. NAD(P)H-FLIM based endogenous biomarker visualization is an effective way to image intrinsic metabolism as NAD(P)H lifetimes and free: bound ratios change with alterations in metabolic state. The metabolic state of immune cells like microglia can be different from other non-immune cells and the surrounding tissue, and these differences are reflected by the alternations in their lifetime signature.

For fitting-based methods (FBM), where the fitted parameters were exported from SPCImage, we were able to achieve TPR of 0.90 ± 0.03 for five different microglia mixed culture group (Figure 2). However, we got some false positives with FNR being 0.09 ± 0.03 and FDR being 0.33 ± 0.10. For clinical application the false positive need to be reduced in future studies. We expect this number will be improved by increasing the number of training samples with more diverse samples matching the realistic use case for *in vivo* imaging. This approach would require a significant amount of data acquisition and processing time as each FLIM image takes 1–2 min to acquire. This FBM approach was also applied to brain tissue sections, which achieved a lower accuracy than cell culture which we expected. The tissue is much more heterogeneous and different fluorescent signature add to the variation in lifetime alternations. Still, we managed to achieve TPR of 0.79 ± 0.08 and PPV of 0.64 ± 0.09 but increased false positive resulted in FNR of 0.2 ± 0.08 and FDR of 0.36 ± 0.09 (Figure 3). The reduction in TPR for tissue can be attributed to several factors. In the mixed glial cell, there were primarily glial cell of which majority of non-microglia cells were astrocyte. The lifetime variation is much smaller in this environment compared to an actual brain tissue where lots of different factors contribute to the NAD(P)H lifetime variation. We also had fewer number of microglia cell for training for the same number of FOVs, which can also contribute to the reduction in accuracy to some extent. Combination of these factors can contribute to the reduction in TPR and increase in FDR.

The second approach, DBM used the decay curve directly as the input training parameter. However, there is shortfall in the accuracy possibly introduced by curve shift between successive imaging, after pulsing of the detectors that requires taking into account Instrument Response Function (IRF). When the testing instances (from mixed glial culture) were reorganized to form image after classification, we obtained a TPR of 0.36 ± 0.09 but a PPV of 0.82 ± 0.15. But we also got an increased FNR of 0.63 ± 0.08 but reduced FDR 0.17 ± 0.15. This method has the lowest TPR but also achieved the best FDR. With more training samples this method could lead to significantly better performance. This method did not yield an acceptable result in tissue as the heterogeneity in the tissue might add to the variation. To make this method more accurate and clinically viable, we need more training datasets as well as take into account the FLIM instrumentation originated variation such as shift in decay and IRF. Moreover, surface-based markers such as CD11b for microglia could result in a better classification compared to Iba1 as they are able to properly identify all of cell body and processes which would reduce false negatives for training. We intend to explore this further in future work, as well as combining advanced deep learning tools such as Convolutional Neural Networks (Krizhevsky et al., 2012) to better predict microglia location using lifetime information and morphological features.

One limitation of our approaches is inherent to the TCSPC approach itself, as TCSPC acquisition takes several minutes to finish a single frame depending on the sample fluorescence intensity. For *in vivo* live acquisitions, the TCSPC approach might be less useful if real-time visualization is required. Frequency domain FLIM (Gratton et al., 2003) acquisition followed by ML could be another approach to overcome acquisition time limitation. Another limitation is the variability of relative shift in the exponential decay, although this can be overcome with the fitting-based method. The FBM already deals with shifts during the fitting process by the fitting software. It could be an issue with the decay-based method where the shift is not taken into account. It might be one of the reasons why the decay-based method did not yield good results with tissue sections. We plan to address the issue with the decay-based approach in tissue sections by using more training data in the future, incorporating the IRF and adding more classification features that consider morphology and/or the intensity of different spectral channels. Other potential exponential curve fitting issues include fitting bias and local minima. One way of overcoming this issue would be avoiding curve fitting by either using raw data (which we demonstrated) and using phasor analysis methods with the TCSPC FLIM data. We ran some preliminary experiment to test the accuracy of the phasor-based approach (TPR 0.41 ± 0.03, PPV 0.49 ± 0.11, FDR 0.51 ± 0.12) and found that false positives were relatively high. But in the future, a hybrid approach where phasors are part of classification features alongside morphology could provide better discrimination for cell type. Another issue we would like to bring to attention is the tissue/cell fixation. All of our testing and training was performed using fixed cell/tissue. While there is some past concern with FLIM imaging with fixed samples for NAD(P)H imaging, our lab has demonstrated recently that, although fixation causes shift in NAD(P)H lifetime values, the metabolic signature and trend are not altered (Chacko and Eliceiri, 2019). For classification for live tissue, new training sample would be required to tune the parameters. Another limitation of our experiment was that we did not identify True Negative (TN) and as a result all the components of confusion matrix and accuracy can’t be determined. For the mixed glial culture, the TNs would be the non-microglia glial cell. But it is more complicated in brain tissue to define TN as the brain tissue is heterogeneous and consist of components that are challenging to define in terms of TN. One last limitation would be the variability of NAD(P)H lifetimes from sample to sample. Based on the microenvironment, the NAD(P)H lifetime can vary even though the cells/tissues are treated similarly. One way to overcome this limitation would be to train with larger datasets with similar treatments.

## Conclusion

We have demonstrated a novel machine learning based approach that can use FLIM data to identify microglia based on NAD(P)H lifetime parameters. We have successfully shown the effectiveness of the method in both cells and tissue slices and achieved close to 90% True Positive Rate and moderately low False Discovery Rate. Additionally, we have shown that the decay can be used to directly identify microglia using ANN without exponential curve fitting. This approach can be further enhanced to calculate lifetimes and other parameters from lifetime decay data directly using machine learning.

## Data Availability Statement

The raw data supporting the conclusions of this article will be made available by the authors, without undue reservation.

## Ethics Statement

The animal study was reviewed and approved by the University of Wisconsin-Madison Institutional Animal Care and Use Committee.

## Author Contributions

MS and KE conceptualized the technique and application. MS, KC, JO, JCW, JJW, and KE designed the experiments. KC and JO performed the biology experiments. MS wrote the code for ANN and performed the data analysis. MS, JJW, and KE wrote the manuscript. All authors contributed to the article and approved the submitted version.

## Conflict of Interest

The authors declare that the research was conducted in the absence of any commercial or financial relationships that could be construed as a potential conflict of interest.
